# Phylogeography and population genetic structure of red muntjacs: evidence of enigmatic Himalayan red muntjac from India

**DOI:** 10.1186/s12862-021-01780-2

**Published:** 2021-03-23

**Authors:** Bhim Singh, Ajit Kumar, Virendra Prasad Uniyal, Sandeep Kumar Gupta

**Affiliations:** Wildlife Institute of India, Chandrabani, Dehradun, 248001 UK India

**Keywords:** Phylogeography, Red muntjacs, *M. aureus*, Mitogenome, Microsatellite, Evolutionary Significant Unit

## Abstract

**Background:**

Identifying factors shaping population genetic structure across continuous landscapes in the context of biogeographic boundaries for lineage diversification has been a challenging goal. The red muntjacs cover a wide range across multiple vegetation types, making the group an excellent model to study South and Southeast Asian biogeography. Therefore, we analysed mitogenomes and microsatellite loci, confirming the number of red muntjac lineages from India, gaining insights into the evolutionary history and phylogeography of red muntjacs.

**Results:**

Our results indicated the Northwestern population of red muntjac or the Himalayan red muntjac (*M. aureus*) in India as genetically diverse and well-structured, with significant genetic differentiation implying a low level of gene flow. The phylogenetic, population genetic structure, as well as species delimitation analyses, confirm the presence of the lineage from Western Himalayan in addition to the previously identified red muntjac lineages. Relatively low genetic diversity was observed in *M. aureus* compared to *M. vaginalis*, *M. malabaricus* and *M. muntjak.* The *M. aureus* and *M. vaginalis* lineages have split during the late Pleistocene, ~ 1.01 million years ago (Mya), making *M. aureus* the youngest lineage; whereas, *M. malabaricus* split earlier, ~ 2.2 Mya and appeared as the oldest lineage among red muntjacs.

**Conclusions:**

Pronounced climate fluctuations during the Quaternary period were pivotal in influencing the current spatial distribution of forest-dwelling species’ restriction to Northwestern India. Our finding confirms the distinct Himalayan red muntjac (*M. aureus*) within the red muntjac group from Northwestern India that should be managed as an Evolutionary Significant Unit (ESU). We recommend a reassessment of the conservation status of red muntjacs for effective conservation and management.

**Supplementary Information:**

The online version contains supplementary material available at 10.1186/s12862-021-01780-2.

## Background

The genus *Muntiacus* belongs to tribe Muntiacini within the family Cervidae. It is widely distributed throughout South and Southeast Asia [1], and exhibits extreme variations in chromosome numbers [2, 3]. The taxonomic classification and validation of species and subspecies is still controversial. Mattioli classified red muntjacs as single species *Muntiacus muntjak* comprising ten subspecies [4]. Grubb and Groves recognized six species and two subspecies of *Muntiacus* using geographical distributions and morphological characters [5]. International Union for Conservation of Nature (IUCN) has provisionally adopted two species of *Muntiacus: M. vaginalis* (Northern or Indian Red Muntjac) and *M. muntjak* (Southern Red Muntjac). While the former is widely distributed from northern Pakistan to most of India, Nepal, Bhutan, Bangladesh to southern China with Hainan and south Tibet, and into Myanmar, Thailand, Lao, PDR, Vietnam, Cambodia, the latter is limited to the Thai–Malay peninsula, Java, Bali, Lombok, Borneo, Bangka, Lampung and Sumatra [6]. However, the exact southern range limit of Muntjacs in the Thai–Malay peninsula remains unclear.

For highly adapted mammals such as red muntjac, whose extensive distribution ranges are linked to different political boundaries, contemporary genetic variation and population structure may be shaped by both natural and anthropogenic factors [7]. With the increasing number of studies on muntjacs, the number of newly recognized *Muntiacus* specie*s* is also increasing. For example, based on the differences in skin color, skull and antler morphology, a new endemic species from Borneo (Bornean yellow muntjac or *M. atherod*) was described [8], after which the Gongshan muntjac (*M. gongshanensis*) was described from Southwest, China and northern Myanmar [9, 10]. Previously, the Putao muntjac (*M. putaoensis*) from Myanmar [11], Small blackish muntjac (*M. truongsonensis*) from Central Vietnam [12], and Roosevelt’s barking deer (*M. rooseveltorum*) from Vietnam [13] have been confirmed with molecular studies. In a recent study, the complete mitogenome sequences indicated the presence of three distinct maternal lineages across the distribution range of red muntjacs: Srilankan red muntjac from Western Ghats, India and Sri Lanka; Northern red muntjac in Northern India and Indochina; and Southern red muntjac found in Sundaland [14]. Distinct morphological and genetic characters are a center of attraction to study the mystifying red muntjacs. It is of urgent importance when anthropogenic activities such as habitat fragmentation and destruction with poaching have drastically influenced the population size, distribution ranges and population genetic structure of several deer species in the last few centuries [15–17]. The Indian subcontinent sustains a diverse ecosystem that supports high faunal richness and diversity, but very little is known about the species diversification and evolutionary history and the role of geo-climatic changes during the Late Pleistocene [18, 19]. The late Pliocene to early Pleistocene witnessed dramatic climatic shifts in South and Southeast Asia, which led to geographical subdivision with contraction of habitats, influencing the distribution of contemporary species. The persistence of rainforests in high elevation areas led to many refugia populations inhabiting the high mountainous region [20–22]. The wide distribution range of the Northern red muntjac across multiple biogeographic zones makes it an excellent model to study the biogeography and diversification of red muntjacs in South and Southeast Asia.

The Northern red muntjac is currently listed as “Least Concern” in the IUCN Red List and protected under Schedule III of the Indian Wild Life (Protection) Act, 1972. Extensive genetic characterization of Northern red muntjac from India can depict population boundaries and genetic structuring for its appropriate classification and formulation of management strategies. Recently, Martins et al. (2017) investigated the geographic distribution of mtDNA lineages among red muntjac populations using museums, zoos, and opportunistically collected samples from Vietnam, Laos, and Peninsular Malaysia [14]. Due to museum samples, the complete mitogenomes from this study contained several ambiguous nucleotides in most of the sequences. Martins et al. (2017) also suggested extensive sampling to unveil taxonomic uncertainties within red muntjacs with nuclear data analysis to examine barriers to gene flow [14].

Moreover, Groves et al. [5] and Martins et al. [14] have previously suspected the presence of a distinct lineage from Northwestern India, Central India, and Myanmar. Therefore, to investigate the phylogeography and molecular ecology of red muntjacs from India, we combined newly generated complete mitogenome sequences with data by Martins et al., [14] to gain insights into the evolutionary history and phylogeographic pattern of red muntjacs. We further employed nuclear markers to substantiate the characterization of the Indian muntjacs.

## Results

### Genetic diversity

The 16 generated mitogenomes sequences of red muntjac were deposited in GenBank (MT671398-MT671408; MT758349-MT758353). To elucidate the phylogenetic relationships, we included four sequences of *M. vaginalis* from Singh et al. [23] and 32 sequences of Northern, Southern, and Srilankan muntjacs from Martins et al. [14]. The accession numbers and details for each sample are provided in the Additional file 1: Table S1. All complete mitogenome sequences grouped into four genetically distinct clusters and represented individual haplotypes with haplotype diversity Hd = 1, indicating maternal unrelatedness among the red muntjac samples (Table 1). The nucleotide diversity of *M. vaginalis* (Mainland red muntjac) was *π* = 0.0044 (s.d. = 0.0003), *M. aureus* (Himalayan red muntjac) was *π* = 0.0016 (s.d. = 0.0002), *M. malabaricus* (Western Ghats & Srilankan red muntjac) was *π* = 0.005 (s.d. = 0.0008) and *M. muntjak* (Southern or Sundaland red muntjac) was *π* = 0.0109 (s.d. = 0.0006). The overall nucleotide diversity among red muntjacs was *π* = 0.0203 (s.d. = 0.001). Among all red muntjacs, the lowest number of segregating sites were found in *M. aureus* (S = 67), whereas it was high in *M. muntjak* (S = 658). Tajima’s D and Fu’s FS test were non-significantly different from zero (P > 0.01), which indicated that red muntjac populations had not undergone an expansion.

**Table 1:** Summary of genetic diversity in red muntjacs populations based on microsatellites and complete mitochondrial DNA

Population/Region	Mitochondrial DNA							Microsatellites					
	n	S	H	Hd	π	Tajima’s D^*a*^	Fu’s *F*s^*a*^	n	Na	*Ar*	*H*_o_	*H*_E_	*F*_*IS*_
*M.aureus*	6	67	6	1.00	0.001	− 0.49	0.46	18	7.11 ± 0.73	4.52	0.66 ± 0.071	0.73 ± 0.03	0.131
*M.vaginalis*	23	415	23	1.00	0.004	− 1.33	− 2.7	19	9.33 ± 0.86	5.79	0.62 ± 0.04	0.81 ± 0.02	0.269
*M. malabaricus*	6	198	6	1.00	0.005	0.48	1.9	5	6.11 ± 0.56	6.11	0.75 ± 0.06	0.76 ± 0.04	0.096
*M. munjak*	17	658	17	1.00	0.01	− 0.37	− 0.01	–	–	–	–	–	–

*n* number of samples, *S* segregating sites, *H* haplotype, *Hd* haplotype diversity, *π* nucleotide diversity, *Na* number of alleles, *Ar* allelic richness, *H*_O_ observed heterozygosity, *H*_E_ expected heterozygosity, F_*IS*_ inbreeding coefficient, ^a^all P-values > 0.01 (not significant)

The genetic diversity of Indian red muntjacs was calculated using nine microsatellite markers (Table 1). The selected microsatellite markers showed high polymorphic information content (PIC > 0.5) with a mean value of 0.831; therefore, all used loci were found to be informative. All the loci significantly deviated from Hardy–Weinberg equilibrium (HWE) and no linkage disequilibrium (LD) was detected (P > 0.05). No evidence of a large allele drop out was observed, while null alleles at each locus were low in frequency (less than 0.10 per population). The mean number of alleles (Na) in *M. vaginalis, M. aureus* and *M. malabaricus* were 9.33, 7.11 and 6.11 respectively with highest allelic richness (*Ar*) in *M. malabaricus* (*Ar* = 6.11) and lowest in *M. aureus* (*Ar* = 4.52). The observed heterozygosity (*H*o) and expected heterozygosity (*H*_E_) in *M. aureus* were *H*o: 0.666; *H*_E_: 0.730; for *M. vaginalis, H*o: 0.620; *H*_E_: 0.810; whereas, in *M. malabaricus*, it were *H*o: 0.756, *H*_E_: 0.760. The mean inbreeding coefficient (*F*_IS_) value for all the red muntjac populations were greater than zero (ranges between 0.096 and 0.269), indicating a heterozygote deficiency (Table 1), which may be attributed due to the Wahlund effect and population not being in HWE.

### Phylogeography of red muntjacs

**Fig. 1:**
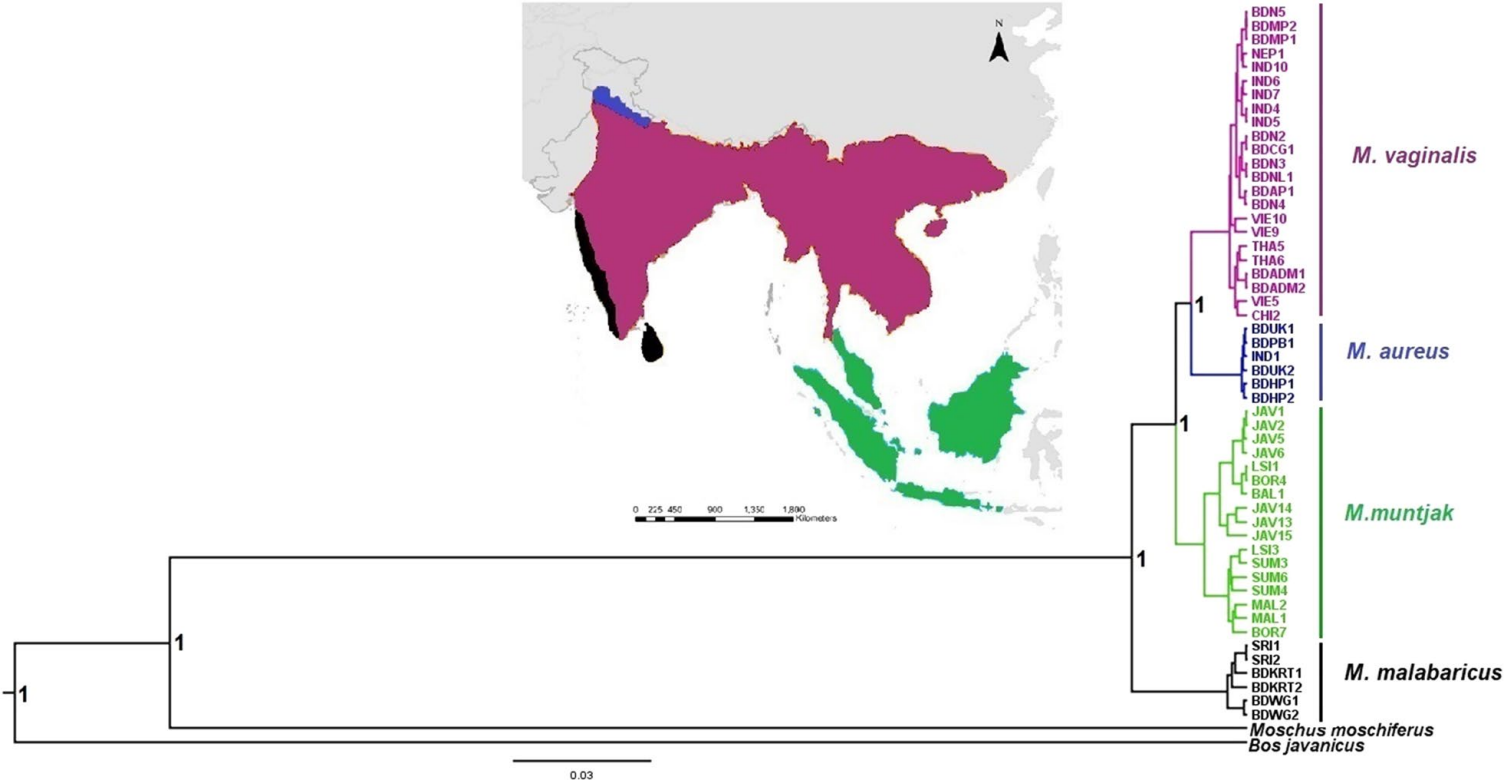
Bayesian inference (BI) of phylogenetic tree for red muntjac based on complete mitochondrial DNA. Numbers on clades indicate posterior probability (PP) for the node. The distribution ranges of different lineages are represented by specific colors in distribution map corresponding to colored clades in a tree topology

**Fig. 2:**
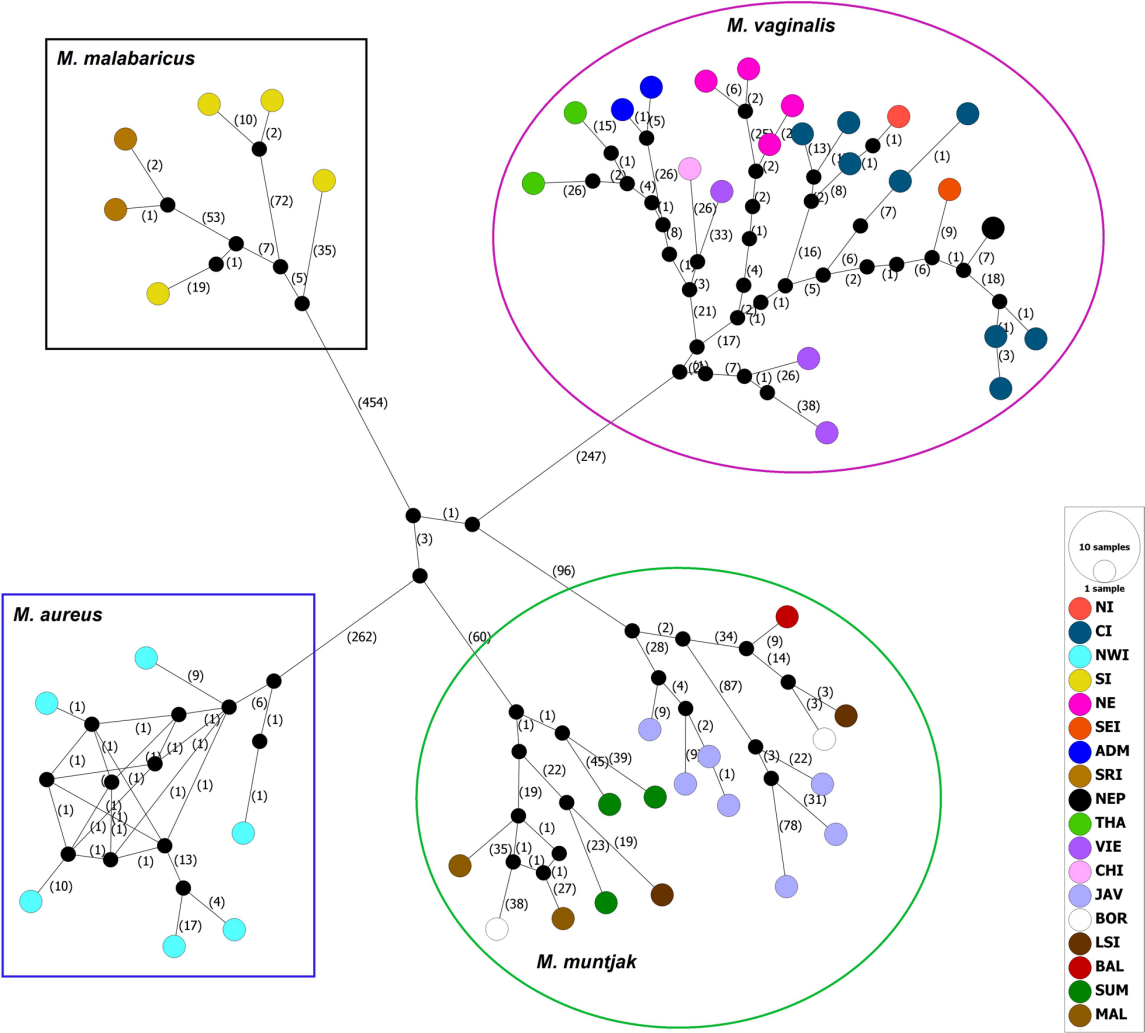
A median-joining (MJ) network of full mitogenomes of red muntjac lineages. The numbers in bracket (n) represent the number of mutations seperating the haplotypes. The size of each circle indicates the relative frequency of the corresponding haplotype in the whole dataset

The Bayesian consensus tree showed that all sequences of red muntjac clustered into four major clades (Fig. 1). Clade-I consisted of Mainland red muntjac lineages (*M. vaginalis*), comprising individuals from Northern to Central India, Eastern to Northeastern India, Nepal, Southeast Asia, and Andaman & Nicobar Islands. Clade-II comprised the individuals from the Northwestern part of India (*M. aureus*) (i.e., Uttarakhand, Punjab, and Himanchal Pradesh) and Clade-III comprised the sequences from Sunda (*M. muntjak*), mainly from Sumatra, Malay Peninsula, Lombok, Borneo, Java, and Bali’s Islands. Clade-IV consisted of individuals from Western Ghats of Southern Indian and Sri Lanka (*M. malabaricus*). The median-joining (MJ) network of all recognized sequences from India, Southeast Asia Sundaland, and Sri Lanka strongly supported the phylogenetic results, indicating the existence of four geospatial populations from its distribution ranges (Fig. 2). In India, we found three evident clades representing populations of *M. vaginalis* (Clade-I), *M. aureus* (Clade-II)*,* and *M. malabaricus* (Clade-IV). The phylogenetic and median-joining analyses showed the presence of two sub-groups in the Sunda of Southern red muntjac. It is also noteworthy that the mainland red muntjacs from India exhibited different genetic signature and showed structuring with respect to other populations in Southeast Asia and Andaman & Nicobar Islands.

### Estimating genetic divergence

**Fig. 3:**
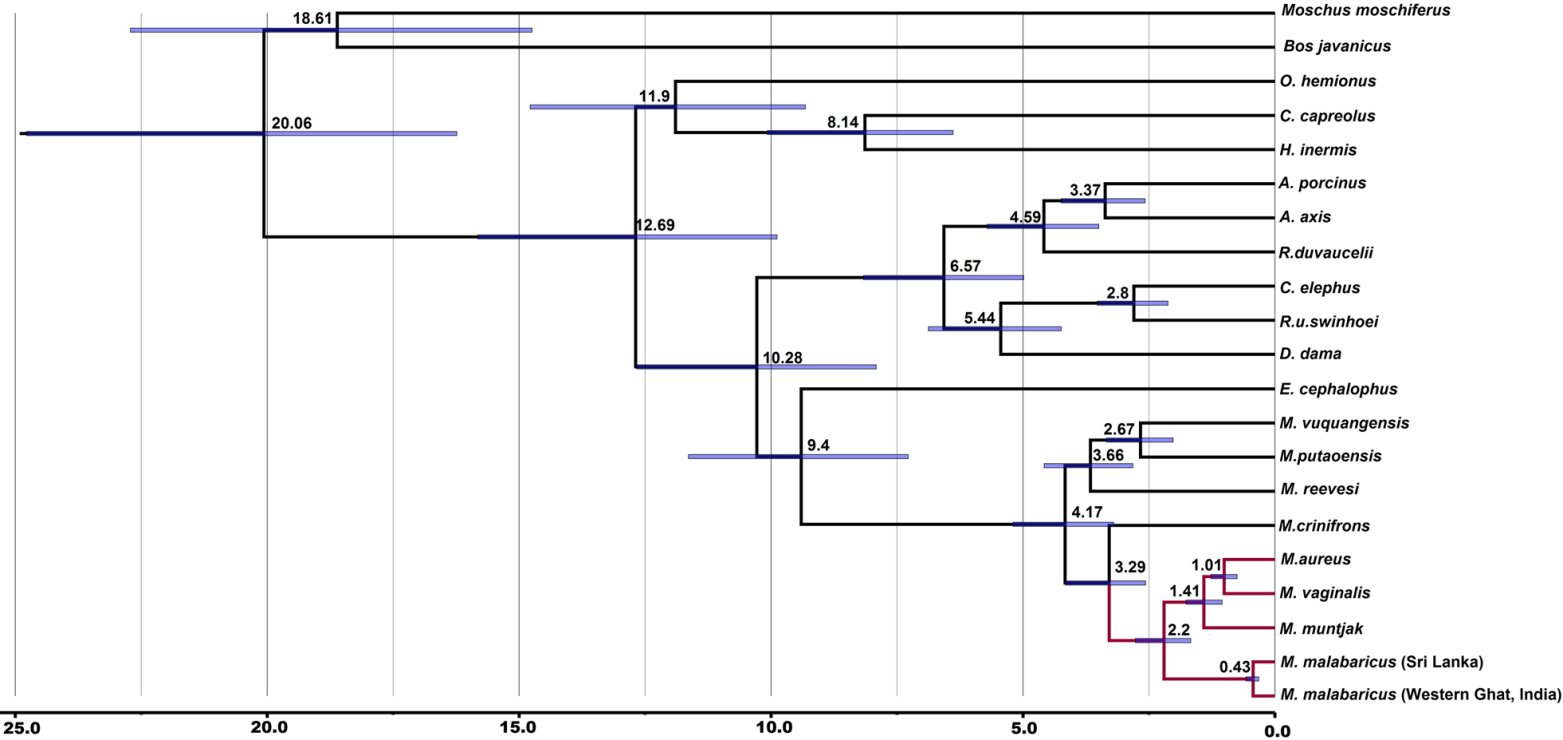
Divergence time estimation based on maximum credibility tree using complete mitogenome generated from BEAST analysis

We calibrated the root age (TMRCA of Bovidae and Moschidae) to 18 ± 2 Mya (CI_95%_: 14.74–22.94) and the split between Cervidae and Bovidae + Moschidae was set at 17.2 ± 2 Mya (CI_95%_: 16.04–24.08). Our divergence results suggested that the split between the red muntjacs and black muntjac (*M. criniforns*) occurred in the Late Pliocene, around 3.29 Mya (CI_95%_: 2.57–4.16). Within red muntjac, group diversification started during the Pleistocene. The *M. malabaricus* split earlier ~ 2.2 Mya (CI_95%_: 1.67–2.77), and thereafter, the clade of *M. muntjak* of Sunda split around 1.4 Mya (CI_95%_: 1.05–1.76). Within the northern lineages, the split between the *M. vaginalis* and *M. aureus* was estimated to have occurred at ~ 1.01 Mya (CI_95%_: 0.75–1.27) (Fig. 3). Our analysis indicated the Northwestern red muntjac to be the youngest among the red muntjac.

### Species delimitation

**Fig. 4:**
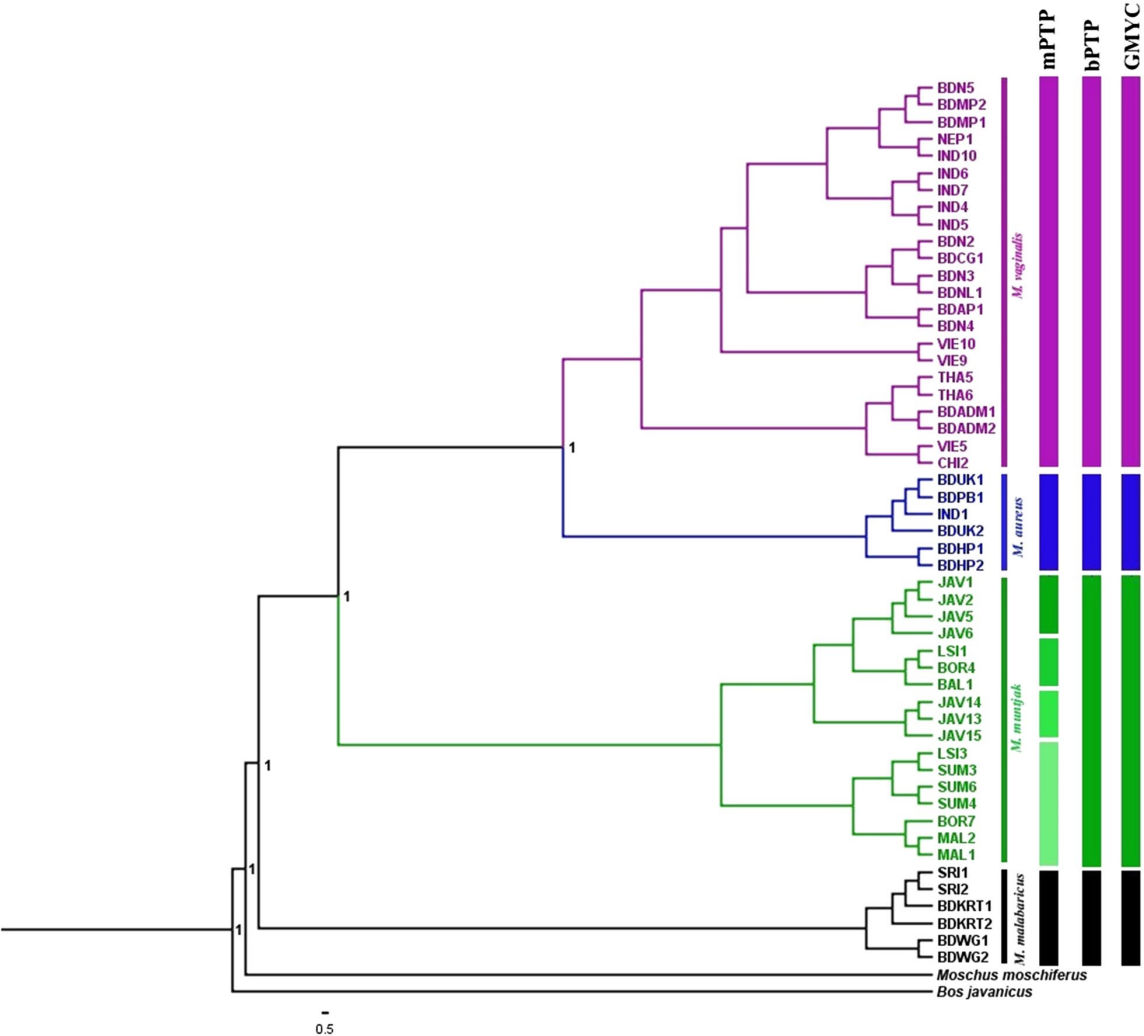
Bayesian phylogeny based on complete mitochondrial genomes representing the result of three different molecular species delimitation methods. Multi-rate Poisson Tree Processes (mPTP), Bayesian Poisson Tree Processes (bPTP) and Generalized Mixed Yule-Coalescent (GMYC)

The bPTP and GMYC analyses indicated four species in our dataset, whereas the mPTP analysis indicated seven. All analyses delimited the same taxonomic units as inferred from BEAST phylogenetic analysis, namely: *M. vaginalis*, *M. aureus*, *M.muntjak* and *M. malabaricus*, while the mPTP model additionally expanded the *M. muntjak* group from Sundaland into 4 subsets (Fig. 4). The result of bPTP and GMYC analyses supported the previously recognized taxonomic subdivisions, also corroborated by our analyses.

### Population genetic structure and genetic differentiation

**Fig. 5:**
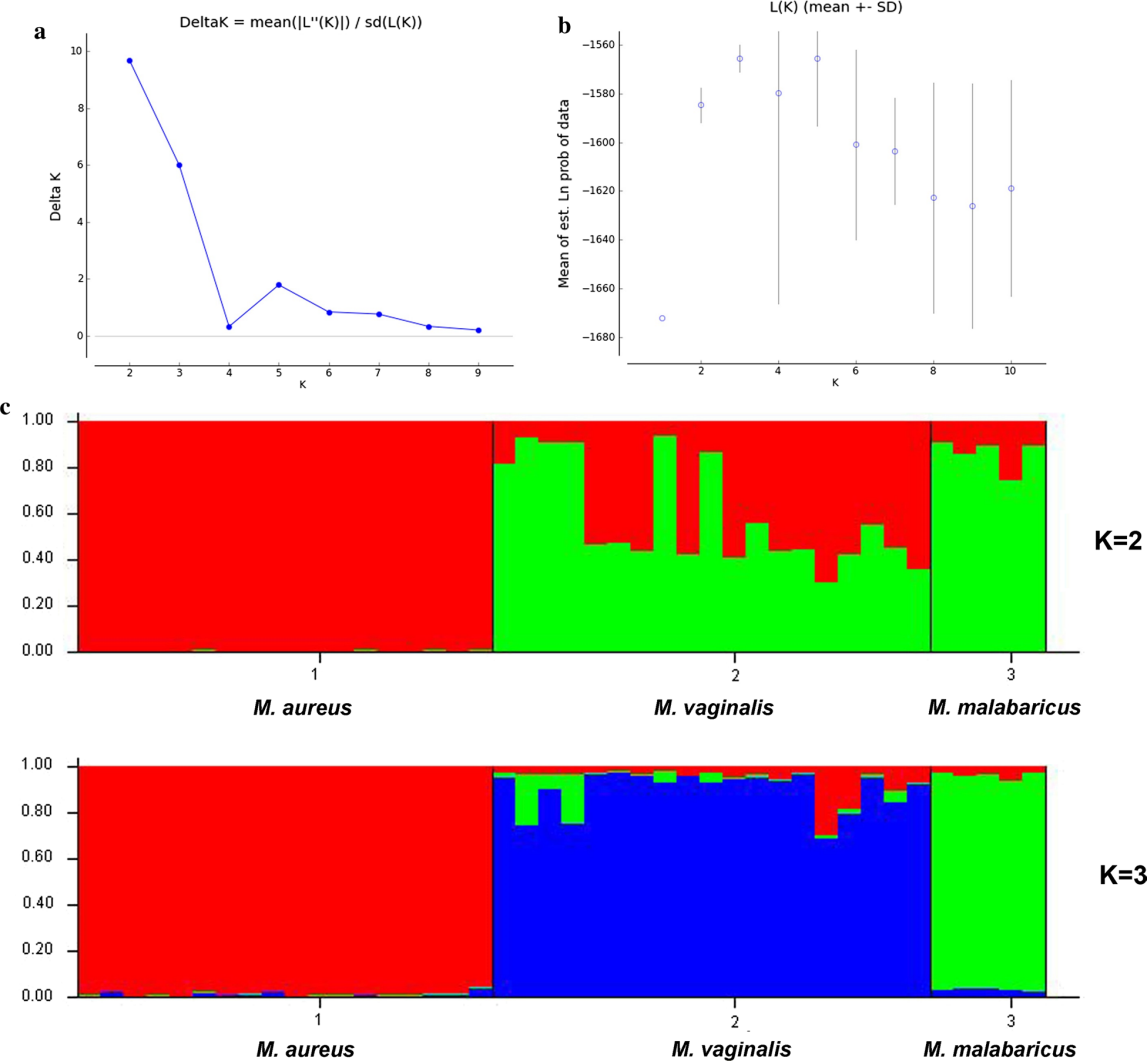
Results of genetic clusters inferred from Structure 2.3.4. **a** Delta K values with respect to K **b** Mean of estimates Ln probability of data with respect to K. **c** Barplot indicating the genetic structure at K = 2 and K = 3, showing the population assignments for each individuals of *M. aureus*, *M. vaginalis* and *M. malabaricus*, respectively. The Y axis is depicting the proportion derived from each cluster

**Fig. 6:**
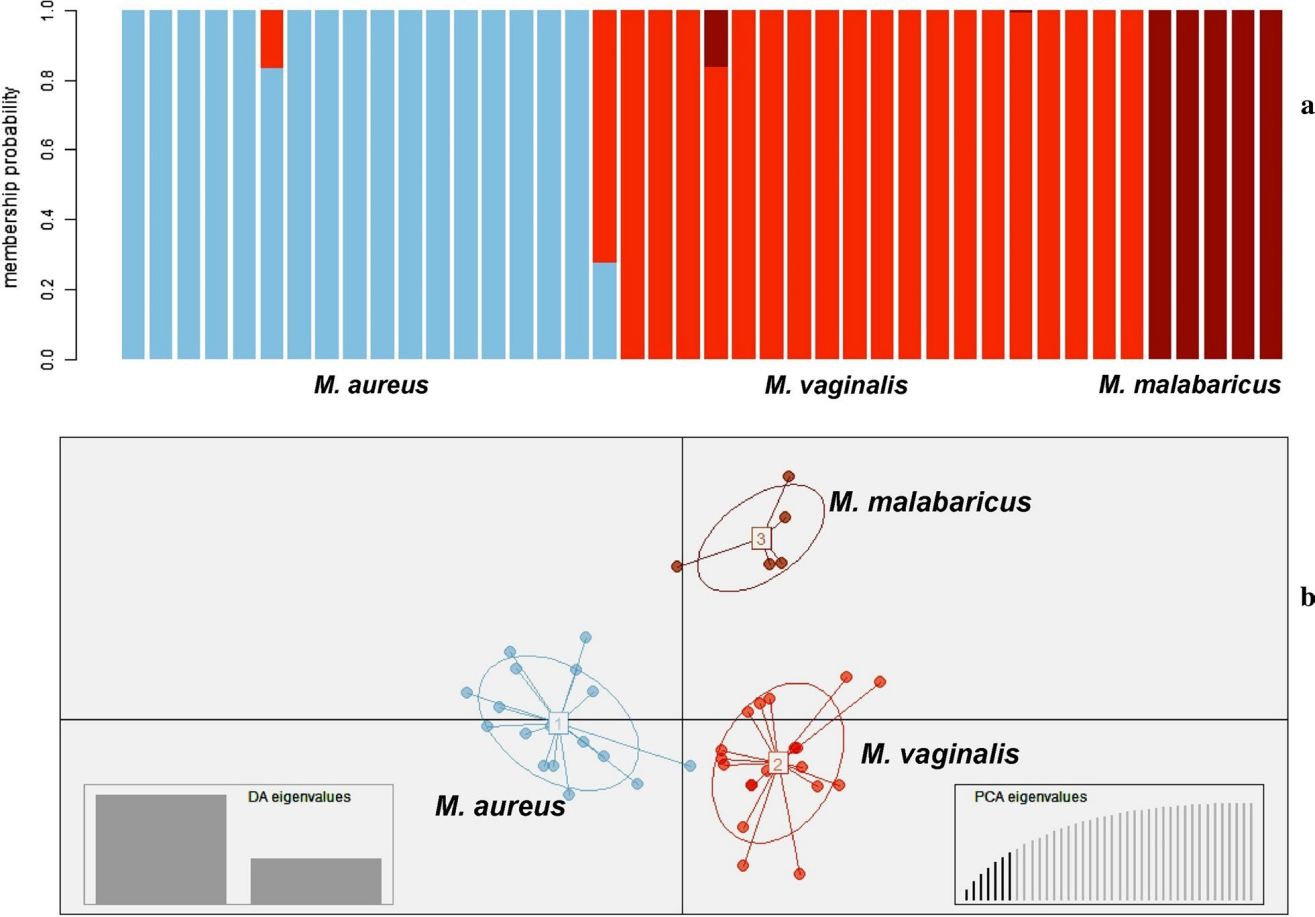
The bar plot results show genetic clustering implemented in Discriminant Analysis of Principal Components (DAPC) (**a**). Each column along the X-axis represents one red muntjac individual. The Y-axis represents the assignment of the membership probability of each individual. Scatterplots of DAPC using a hierarchical islands model and shown by different colors and inertia ellipses (**b**). The DA and PCA eigenvalues of the analyses are displayed in the inset

The Bayesian clustering analysis identified the highest ΔK when *K* was set at 2 (Mean LnP(K) = − 1584.64; ΔK = 9.6) under the admixture model. As recommended by Evanno et al. (2005) interpret *K* with caution, we further analyzed accordingly to identify the possible hidden substructure for each predefined cluster [24]. Therefore, we adopted the second-highest value of *K* = 3 (Mean LnP(K) = − 1565.47; ΔK = 5.9), where most individuals were assigned to three different clusters, which differentiated among Northwestern (*M. aureus*), Mainland (*M. vaginalis*), and Western Ghats population (*M. malabaricus*) (Fig. 5). Although the genetic structure of the Mainland population indicated some alleles from the Himalayan and Western Ghats populations, none of the alleles were found vice-versa. The multivariate Discriminant Analysis of Principal Components (DAPC) (Fig. 6) and factor correlation analysis (FCA) (Additional file 1: Figure S1) also supported the Bayesian clustering method that differentiated the populations into three genetic clusters. We found strong concordance between microsatellite and mitogenome data using different Bayesian and non-Bayesian clustering methods used in this study.

**Fig. 7:**
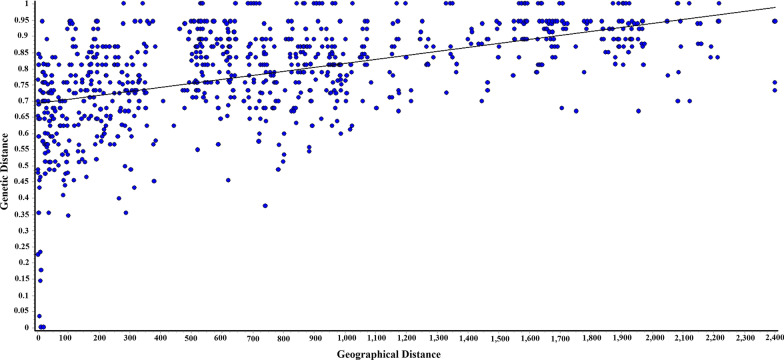
Correlation of genetic and geographical distance in kilometer between red muntjac population using microsatellite data (Mantel test statistic, *rM* = 0.513; *P* = 0.0009)

The analysis based on pairwise *F*_ST_ for red muntjac using complete mitogenome demonstrated significant genetic differentiation of *M. vaginalis* from *M. aureus* (0.0208) and *M. malabaricus* (0.0396). The genetic differentiation of *M. aureus* from *M. vaginalis* and *M. muntjak* was almost similar (0.026), whereas a comparatively high level of differentiation was observed between *M. muntjak* and *M. malabaricus* (0.039). A parallel pattern was observed in microsatellite analysis where the observed genetic differentiation between the *M. aureus* and the *M. vaginalis* was 0.062, and *M. malabaricus* was 0.105, whereas *M. vaginalis* to *M. malabaricus* was 0.080 (Table 2). We also calculated genetic differentiation with other *Muntiacus* species and recovered a comparatively low level of genetic differentiation between red muntjac lineages with *M. criniforns* (0.056 to 0.057) than with other species such as *M. reevesi* (0.065 to 0.068)*, M. vuquangensis* (0.060 to 0.068) and *M. putaoensis* (0.067 to 0.069)*.* The spatial genetic analysis revealed a significant correlation between the pairwise genetic distances among geographical subsets and geographical distances (Mantel test statistic, *rM* = 0.513; *P* = 0.0009, Fig. 7). This pattern of isolation by distance (IBD) was strongly influenced by the genetic differentiation and major geographical distance between the red muntjac lineages.

**Table 2:** Genetic differentiation among red muntjac and other Muntiacus species

Species	1	2	3	4		5	6	7	8
*M. vaginalis*	–	**0.062**	**0.080**						
*M. aureus*	0.0208	–	**0.105**						
*M. malabaricus*	0.0396	0.0401	–						
*M. muntjak*	0.0269	0.0264	0.0399	–					
*M. criniforns*	0.0557	0.0572	0.0562	0.0571	–				
*M. reevesi*	0.0668	0.0657	0.0682	0.0685	0.0651		–		
*M. vuquangensis*	0.0679	0.0683	0.0672	0.0681	0.0665		0.0606	–	
*M. putaoensis*	0.0693	0.0690	0.0676	0.0695	0.0692		0.0624	0.0486	–

The pairwise *F*_ST_ values based on the complete mitogenome and microsatellite loci (in bold) are shown above and above the diagonal, respectively

## Discussion

The study brings together the extensive analyses of complete mitochondrial and microsatellite loci variation to understand the Quaternary climatic fluctuations and geological events on the probable influence on the demographic pattern and population genetic structure among the red muntjac groups. Previous phylogeographic studies of red muntjac showed a clear division between Northern and Southern lineages, indicating physical barriers to gene flow resulting from extremely dry climatic conditions caused by global ice advances [14]. As the taxonomy of muntjacs is considerably fragile and still debated [5], ongoing research aims to resolve the phylogenetic complexities to elucidate the exact number of lineages. The population genetic studies on red muntjacs will act as definitive tools to understand the lineage diversification, genetic structuring, and diversity, resulting in developing appropriate conservation and management strategies for this enigmatic species. Previously, Martins et al. (2017) reported three mitochondrial lineages: The Srilankan red muntjac (*M. malabaricus*), Northern red muntjac (*M. vaginalis*) and Southern red muntjac (*M. muntjak*) [14]. The present study on genetic structure and differentiation among red muntjac populations exhibited four genetically distinct lineages from its geographical distribution range in South and Southeast Asia. Structure, DAPC and FCA analyses supported the result with three genetic clusters of red muntjac from India. The species delimitation methods supported taxonomic resolution findings among *Muntjacs*, corroborating the divergence between *M. vaginalis* and *M. aureus* as distinct taxonomic units while grouping the *M. malabaricus* from the Western Ghats and Sri Lanka within a single taxonomic unit. We detected clear genetic signatures of the Northwestern and Western Ghats lineages of red muntjac, whereas few alleles of these lineages were observed in the Indian mainland red muntjac indicating historical gene flow, while the current populations have undergone well-defined structuring and differentiation. Congruent to their distribution pattern, all red muntjac populations exhibited high haplotypes and microsatellite diversity with significant divergence among them. In addition, comparatively lower *Ar* and *H*_E_ were observed in *M. aureus*, which could be attributed to a limited distribution range. The mitogenomic data estimated the split between the Northwestern and Mainland lineages to late Pleistocene ~ 1.01 Mya (CI_95%_: 0.75–1.27), indicating the most recent split among red muntjacs. The Himalayan red muntjac inhabits the foothills of the Himalaya in the Northwestern part of the Indian states of Uttarakhand, Himachal Pradesh, and Punjab. The Northwest lineage was suspected to be distributed in Northwestern and Central India and Myanmar [5]. The presence of distinct genetic lineage from the Northwestern Indian region was also speculated by Martins et al. based on a single sequence (*IND1*) from Himachal Pradesh Province that formed a distinct placement [14]. However, due to the unavailability of sufficient sample size, explicit depiction of the lineage could not be done. Hence, we confirmed the previously suspected hypothesis and provided molecular evidence for the Northwestern lineage from India.

The rise of anthropogenic activities in the late Quaternary was a key factor that changed the global biodiversity pattern [21]. Qinghai-Tibetan Plateau (QTP) upliftment played a significant role in faunal and floral diversification and evolution in Himalayan ranges [25]. The upliftment of the Himalayas followed by the Plio-Pleistocene glaciation led to the evolution of high altitudinal elements shaping biogeographic evolution in the Indian Himalayan region [18]. The Himalayan region enabled allopatric speciation through geographic isolation and adaptive diversification across altitudinal gradients [26, 27]. This diversification was also driven by pre-adapted lineages immigrating and undergoing subsequent speciation [28, 29]. Long-term isolation in fragmented habitats restricted gene flow and caused genetic divergence [30–32] that contributed to the evolution of genetically distinct lineages [33]. It was followed by rapid civilization in the Indo-Gangetic plain of North India, causing extensive destruction of natural habitat, altering plants and animals’ ecological and distributional patterns [18].

In India, the Western Ghats lineage was genetically more diverse than the Mainland, Northwestern and Sundaland populations. The phylogenetic analysis indicated that the Srilankan red muntjac (*M. malabaricus*) is the oldest red muntjac lineage showing genetic similarity with India’s Western Ghats population. The genetic divergence result suggested that the Srilankan red muntjac split from the mainland lineage during Pleistocene ~ 2.2 Mya when the climatic condition was quite complex in India [14]. Despite the present biogeographic separation between Southern India and Sri Lanka, both populations are genetically similar at the mitogenomic level. A similar genetic relationship was observed in *Paradoxurus* (Palm civets), where Southern India and Sri Lanka clustered with each other [22]. The Western Ghats and Sri Lankan lineages’ common origin might be due to the historical connectivity between these two landscapes. Thereafter, changes in sea levels may have led to isolation causing local endemism [22, 34, 35]. In India, the discontinuity of Western Ghats with mainland population could be due to unfavorable habitat conditions that culminated in isolated patches forming the refugia population inhabiting Western Ghats biodiversity hotspot [36]. The barrier formed by India’s central dry zone restricted the gene flow between the Western Ghats and the rest of the Indian population of red muntjac. The restriction in species distribution is probably due to pronounced climate fluctuation in the last glacial maxima that caused the contraction of the rainforest, with forest-dwelling species restricted to the available habitats in high elevation areas [21, 22].

The mainland red muntjac (*M. vaginalis*) is distributed in a larger landscape in India (i.e., Chhattisgarh, Odisha, West Bengal, North East India, and Andaman & Nicobar Islands) and Indo-Chinese region (Vietnam, China and Thailand). The Andaman & Nicobar Islands individuals were genetically closer to the population of Indo-Chinese red muntjac. Unsuitable conditions limited the mainland population to small humid forest patches. Interglaciation led to the emergence of forests in drier areas that enabled former distributions to reoccupy and extend the red muntjac range. The Southern red muntjac inhabited the Sudanic region (Java, Sumatra, Bali and Borneo) and the lineage diversification has been described by Martins et al. [14]. The major transition zone between the Indochinese and Sundaic zoogeographic subregion is the Isthmus of Kra located on the Malay/Thai Peninsula that might act as a possible barrier preventing gene flow between populations [20, 37]. It is also speculated that rather than geophysical barrier, repeated rapid sea-level changes may have resulted in species’ isolation [37]. A similar diversification pattern has also been observed in South and Southeast Asian species, like rodents, masked palm civet, common palm civet and *Macaca* spp., where the formation of the contemporary phylogeographic and genetic structure occurred during the same period [22, 38–41]. The Southern red muntjac split around 1.4 Mya from the Indochinese Mainland population. This divergence estimation is congruent with the lineages diversification in Amphibians [42]; Birds [43]; Mammals [37]; bats [44] and Leopard cat [45] that occurred in the Indochinese and Sundaic region. Faunal diversification between the Indochinese and Sundaic regions might have resulted from fluctuation in the Indian summer monsoon [46] and sea level rise [37, 47].

## Conclusions

The genus *Muntiacus* is a useful model for identifying cryptic lineages and studying South and Southeast Asian biogeography. This study highlights the need for taxonomic revision within the red muntjac group and confirmed that the Himalayan red muntjac (*M. aureus*) has recently separated from *M. vaginalis*. We suggest the Himalayan red muntjac lineage to be managed as a distinct evolutionary significant unit (ESUs). There is also a need to reassess the conservation status in the IUCN Red List. Identifying concrete geographic limits of red muntjacs should be supplemented with rigorous and robust sampling in South and Southeast Asia. Hence, we suggest undertaking research based on mitochondrial and microsatellite markers to address red muntjacs' unclear status across their entire distribution range. The study contributes to understanding the large-scale drivers of species and provides insight into the linkage of environmental changes with distribution and niche dynamics. Based on these estimates, we can understand the species’ speciation events in South and Southeast Asia impacted by biogeographical changes during the Late Pleistocene.

## Methods

### Sample collection and DNA extraction

**Fig. 8:**
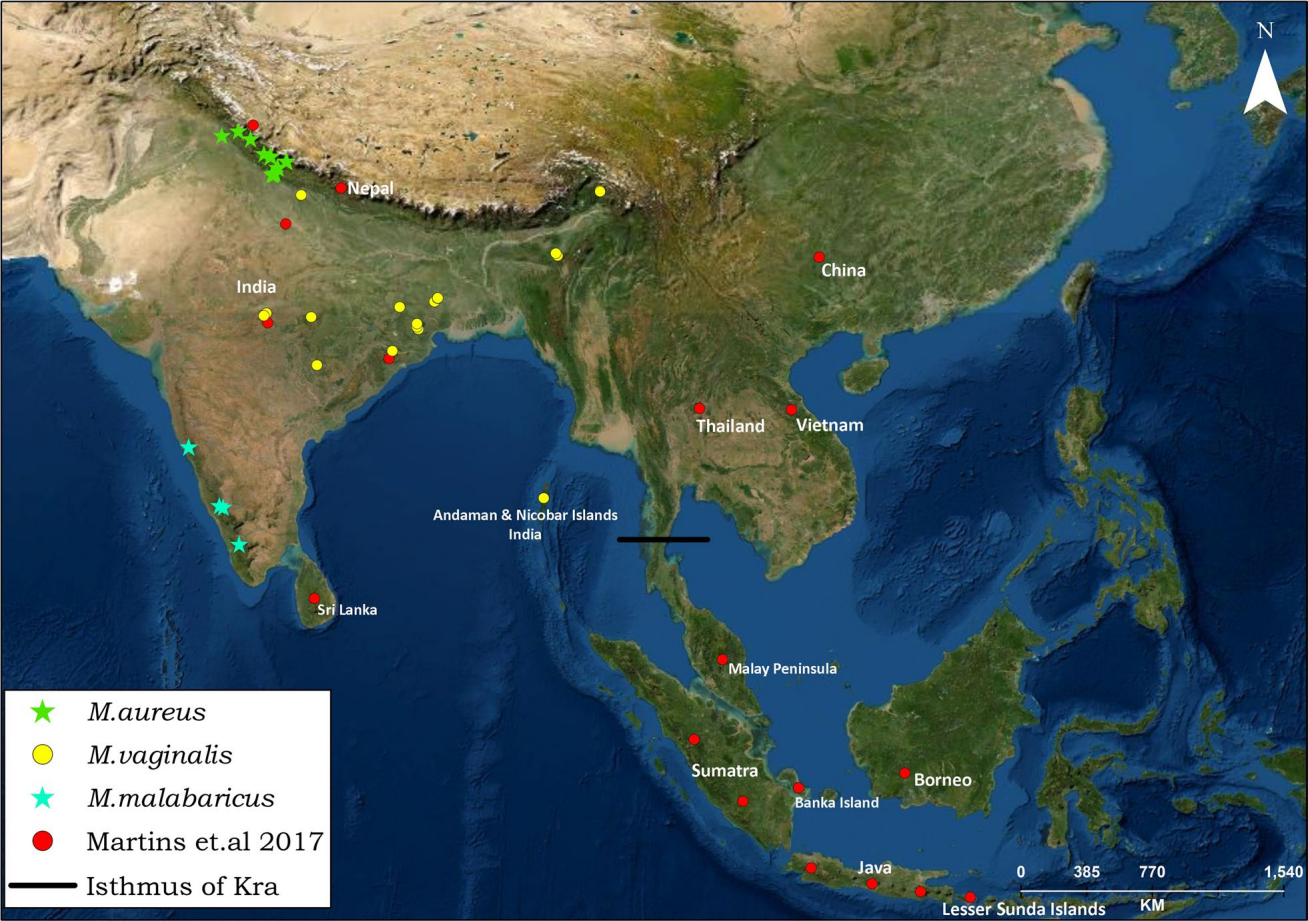
Map depicting the sampling sites of red muntjacs. Yellow dots represent samples collected for the present study; red dots indicate the sampling location used in Martins et al. 2017. The horizontal black line indicates the position of the Isthmus of Kra

We used 42 archival samples of northern red muntjac from different regions of India, including the Northwestern region (NWI = 18), Mainland (localities of north and central) (MLI = 14), northeastern (NEI = 3), Western Ghats (SI = 5) and Andaman & Nicobar Islands (AI = 2) (Additional file 1: Table S2, Table S4 and Fig. 8). Genomic DNA was extracted from tissue and hair samples using modified DNeasy Blood & Tissue kit (Qiagen, Hilden, Germany) protocol, whereas, for antlers and bone samples, we followed Gu-HCl-based silica binding method [48]. These samples were collected from dead animals by the local Forest Department from India's known localities and sent to Wildlife Forensic and Conservation Genetic Cell, Wildlife Institute of India, Dehradun. Since the samples were collected from the dead animals, the Animal Ethics Committee approval was not required. The authors confirm that all experiments were performed following the relevant guidelines and regulations.

### PCR amplification of complete mitogenome and sequencing

Polymerase chain reactions (PCRs) amplifications were carried out in 20 μl volumes containing 10–20 ng of extracted genomic DNA containing, 1 × PCR buffer (Applied Biosystems), 2.5 mM MgCl_2_, 0.2 mM of each dNTP, 5 pmol of each primer, and 5 units of Taq DNA polymerase (Thermo Scientific). We performed PCR amplification using 23 overlapping fragments of complete mtDNA (Additional file 1: Table S3) [49]. Besides, we included the fragments of complete Cytochrome *b* [50] and Cytochrome c oxidase subunit-I gene [51] to increase the overlapping. PCR cycles for DNA amplification were 95 °C for 5 min; followed by 35 cycles of 95 °C for 40 s (denaturation), 54–56 °C (annealing) for 40 s, 72 °C for 50 s (extension) and a final extension of 72 °C for 15 min. The efficiency and reliability of reactions were monitored using controls. The PCR products were electrophoresed on 2% agarose gel and visualized under UV light in the presence of Ethidium bromide dye. The amplified PCR products were treated with Exonuclease-I and Shrimp alkaine phosphatase (USB, Cleveland, OH) for 15 min each at 37 °C and 80 °C, respectively, to remove any residual primer. The purified amplicons were then sequenced bidirectionally using BigDye Terminator 3.1 on an automated Genetic Analyzer 3500XL (Applied Biosystems, Carlsbad, CA, USA).

### Microsatellite loci amplification and genotyping

Nine cross-species microsatellite loci: INRA001 [52], Ca18 [53], BM6506 [54], RT1, RT27, NVHRT48 [55], CelJP27 [56], and T156, T193 [57] were selected for population genetic analysis of red muntjac. Three sets of multiplex panels were carefully assembled based on molecular size and labeled fluorescent dyes of loci. To avoid ambiguity and amplification errors, for each sample, three multiplex PCR were carried out in 10 μl reaction volumes containing 5 μl of QIAGEN Multiplex PCR Buffer Mix (QIAGEN Inc.), 0.2 μM labeled forward primer (Applied Biosystems), 0.2 μM unlabeled reverse primer, and 20–100 ng of the template DNA. PCR cycles for loci amplification were 95 °C for 15 min; followed by 35 cycles of 95 °C for 45 s (denaturation), 55 °C (annealing) for 1 min, 72 °C for 1 min (extension) and a final extension of 60 °C for 30 min. The reliability of reactions was monitored using positive and negative controls. Alleles were resolved in an ABI 3500XL Genetic Analyzer (Applied Biosystems) using the LIZ 500 Size Standard (Applied Biosystems) and analyzed using GeneMaker ver 2.7.4 [58].

### Data analysis

A total of 16 complete mitogenome sequences of red muntjac from five different localities of India were generated (Additional file 1: Table S2 and GenBank accession No. MT671398-MT671408; MT758349-MT758353). Sequences obtained from forward and reverse direction were edited and assembled using SEQUENCHER^®^version 4.9 (Gene Codes Corporation, Ann Arbor, MI, USA). The annotation of complete mitogenome was done using Mitos WebServer [59] and MitoFish [60]. Careful manual annotation was also conducted by sequence alignment with their related homologs sequences or species for ensuring the gene boundaries. Additionally, 36 sequences of northern red muntjac (*M. vaginalis*) (n = 17); southern red muntjac (*M. muntjak*) (n = 17) and Sri Lankan (*M. malabaricus*) (n = 2) muntjac were included from GenBank to cover wide distribution ranges (Additional file 1: Table S1, Fig. 1). The dataset of 52 sequences of red muntjac was aligned using the CLUSTAL X 1.8 multiple alignment programs [61] and alignments were checked by visual inspection. DnaSP v 5 [62] was used to estimate the haplotype (h) and nucleotide (*π*) diversity. The neutrality statistical approaches Tajima’s D [63] and Fu’s *F*s [64] were used to investigate the demographic history of each population using Arlequin ver 3.5 [65].

A total of nine polymorphic loci were used to analyze the 42 red muntjac samples for population genetic studies (Additional file 2: Table S5). The number of loci with genotyping error due to null alleles was assessed using MICROCHECKER 2.2.3 [66]. The CERVUS ver 3.0.6 program [67] was used to estimate the polymorphic information content (PIC), the number of alleles per locus, the observed (*H*_o_) and, expected (*H*_E_) heterozygosity. The *Ar* and mean inbreeding coefficient (*F*_IS_) [68] was estimated using FSTAT ver 2.9.3 [69]. All the loci were checked for HWE in GenAlEx v6.5 [70].

### Phylogeography and population genetic structure

The phylogenetic analysis was conducted in BEAST ver 1.7 [71]. Two outgroup species, *Bos javanicus* (JN632606) and *Moschus moschiferus* (FJ469675) were used to root the phylogenetic tree and the resulting tree was visualized with FigTree v1.4.4 (http://tree.bio.ed.ac.uk/software/figtree/). The spatial distribution of haplotypes was visualized by MJ network created using the PopART software [72]. The genetic distance between lineages was calculated based on the Tamura-3 parameter with a discrete Gamma distribution (TN92 + G) with the lowest BIC score value implemented in MEGA X [73].

To estimate divergence times of red muntjac clades, we inferred genealogies using a strick clock in BEAST ver 1.7 [71]. We performed the dating estimates using 16 sequences downloaded from NCBI, including the species of Cervidae, Muntiacini, Bovidae and Moschidae, i.e., the Chital (*Axis axis*, JN632599), Swamp deer (*Rucervus duvaucelii*, NC020743), Red deer (*Cervus elaphus*, AB245427), European Roe deer (*Capreolus capreolus*, KJ681491), Fallow deer (*Dama dama*, JN632629), Water deer (*Hydropotes inermis*, NC011821), Mule deer (*Odocoileus hemionus*, JN632670), Hog deer (*Axis porcinus, *MH443786), Formosan sambar (*Rusa unicolor swinhoei*, DQ989636), Tufted deer (*Elaphodus cephalophus,*  DQ873526), Chinese muntjac (*Muntiacus reevesi,* NC008491), Giant muntjac (*Muntiacus vuquangensis,* FJ705435), Putao muntjac (*Muntiacus puhoatensis,* MF737190), Black muntjac (*Muntiacus crinifrons,* NC004577), Banteng (*Bos javanicus*, FJ997262), and Musk deer (*Moschus moschiferus*, FJ469675).

Divergence times of phylogenetic clades were calibrated using minimum age of fossil record at two points: one point was calibrated at 18 Mya (normal distribution prior, SD = 2) as the TMRCA (time to the most recent common ancestor) for the split between Bovidae and Moschidae, while the other was set to 17.2 Mya (normal distribution prior, SD = 2) for the split between Cervidae and Bovidae + Moschidae [74, 75]. We used a Yule-type speciation model and the HKY + I + G substitution rate model for tree reconstruction. We conducted two independent analyses, using MCMC lengths of 10 million generations, logging every 1000 generations. All the runs were evaluated in Tracer v. 1.6. The first 10% per run was discarded as burn-in. Maximum credibility trees were obtained with TreeAnnotator (implemented in BEAST ver 1.7 Package) The final phylogenetic tree was visualized in FigTree v.1.4.4 (http://tree.bio.ed.ac.uk/software/figtree/).

To determine the most likely number of the genetic cluster within India, we performed a Bayesian clustering implemented in STRUCTURE 2.3.4 [76]. We analyzed our data using the admixture model and allele frequencies were assumed to be independent with a burn-in of 10,000 followed by 100,000 MCMC (Markov chain Monte Carlo) replications. Ten independent runs were carried out for each cluster set (*K*) from 1 to 10. We also used the ΔK metric to determine the statistically most support number of clusters (*K*) in web interface STRUCTURE HARVESTER [77]. Further, DAPC method was also implemented to identify the number of genetic clusters of the population using the ADEGENET package in R [78]. DAPC is a multivariate and model-free approach that maximizes the genetic differentiation between groups with unknown prior clusters, thus improving populations’ discrimination without requiring the population to be in HWE. FCA was also performed using the GENETIX 4.05 software package [79]. CONVERT 1.31 [80] was used to convert the required input file format. The pairwise *F*_ST_ values among the populations were calculated using GenAlEx ver6.5 [70]. The correlation between the pairwise genetic and geographic distances was performed to detect the pattern of isolation by distance between the disjointed areas, according to Mantel’s test implemented in Alleles in Space 1.0 [81].

### Species delimitation analyses

Species delimitation tests were conducted to validate taxonomic units of *Muntjacs* based on phylogenetic trees derived from whole mitogenome sequences. The analyses were performed using three different approaches: I- Multi-rate Poisson Tree Processes (mPTP) [82]; II- Bayesian Poisson Tree Processes (bPTP) [82]; and III- Generalized Mixed Yule-Coalescent (GMYC) [83].

The mPTP and bPTP analyses were performed on the phylogenetic tree using web server https://mptp.h-its.org and https://species.h-its.org, respectively. Specified parameters for MCMC, thinning, burn-in and seed value were kept as per default settings. The mPTP model employs a fast approach to estimate the maximum likelihood delimitation from an inferred phylogenetic tree, while the bPTP model adds Bayesian support values to the delimited species inferred from the phylogenetic tree. The GMYC model employed the phylogenetic tree after time calibration using HKY + I + G substitution rate model. It delimits species based on likelihood approach fitting branching models within and among species to reconstructed phylogenetic tree. The branch lengths were estimated under a relaxed log-normal clock algorithm in BEAST ver 1.7 [71]. We used MCMC lengths of 10 million generations, logging every 1000 generations. All the runs were evaluated in Tracer v. 1.6. The first 10% per run was discarded as burn-in. Maximum credibility trees were obtained with TreeAnnotator ver1.7 [71]. Single threshold of GMYC model was performed using web server https://species.h-its.org and the output tree is visualized in Tree view [84].

## Supplementary Information

**Additional file 1.** Additional tables and figure.

**Additional file 2: Table S5**. Microsatellite genotype data of Indian red muntjac samples.

## Data Availability

Sequence data are available through NCBI GenBank (Accession No. MT671398-MT671408; MT758349-MT758353).

## Abbreviations

IUCN: International Union for Conservation of Nature
MJ: Median-Joining
TMRCA: Time to a most recent common ancestor
PIC: Polymorphic information content
*H*o: Observed heterozygosity
*H*_*E*_: Expected heterozygosity
DAPC: Discriminant analysis of principal components
FCA: Factor correlation analysis
IBD: Isolation by distance
QTP: Qinghai-Tibetan Plateau
PCR: Polymerase chain reaction
mPTP: Multi-rate poisson tree processes
bPTP: Bayesian poisson tree processes
GMYC: Generalized mixed Yule-Coalescent
