# Supplementary material for: Phylogeography and population genetic structure of red muntjacs: evidence of enigmatic Himalayan red muntjac from India
Source: BMC Ecol Evol. 2021 Mar 23;21:49. doi: 10.1186/s12862-021-01780-2 (PMC7989103; doi:10.1186/s12862-021-01780-2)
Supplement: Supplementary file 1 — Additional file 1. Additional tables and figure. [file 12862_2021_1780_MOESM1_ESM.doc]

**Phylogeography and population genetic structure of red muntjacs: Evidence of enigmatic Himalayan red muntjac from India**

**Bhim Singh, Ajit Kumar, Virendra Prasad Uniyal, and Sandeep Kumar Gupta***

Wildlife Institute of India, Dehradun, India

* Address for Correspondence

| Dr. S. K. Gupta |
| --- |
| Scientist-E |
| Wildlife Institute of India, |
| Chandrabani, Dehra Dun  248 001 (U.K.), India  E-mail: skg@wii.gov.in, skg.bio@gmail.com |
| Telephone: +91-135-2646343 |
| Fax No: +91-135-2640117 |

**Additional file. Table S1: List of complete dataset sequences of red muntjac, with information of Accession number and geographic origin**

| **Sample ID** | **Accession number** | **Geographic Origin** | **Reference** |
| --- | --- | --- | --- |
| BDADM1 | MT671398 | Andaman, India | This study |
| BDADM2 | MT671399 | Andaman, India | This study |
| BDHP1 | MT671400 | Himachal Pradesh, India | This study |
| BDHP2 | MT671401 | Himachal Pradesh, India | This study |
| BDPB1 | MT671402 | Punjab, India | This study |
| BDUK1 | MT671403 | Uttarakhand, India | This study |
| BDUK2 | MT671404 | Uttarakhand, India | This study |
| BDKRT1 | MT671405 | Karnataka, India | This study |
| BDKRT2 | MT671406 | Karnataka, India | This study |
| BDWG1 | MT671407 | Western Ghats, India | This study |
| BDWG2 | MT671408 | Western Ghats, India | This study |
| BDCH1 | MT758349 | Chhattisgarh, India | This study |
| BDNL1 | MT758350 | Nagaland, India | This study |
| BDAP1 | MT758351 | Arunachal Pradesh, India | This study |
| BDMP1 | MT758352 | Madhya Pradesh, India | This study |
| BDMP2 | MT758353 | Madhya Pradesh, India | This study |
| BDN3 | MK050505 | Nagaland, India | Singh et al., 2018 |
| BDN4 | MK050506 | Arunachal Pradesh, India | Singh et al., 2018 |
| BDN5 | MK050507 | Madhya Pradesh, India | Singh et al., 2018 |
| BDN2 | MH547032 | Chhattisgarh, India | Singh et al., 2018 |
| IND1 | KY052109 | North India | Martins et al.,2017 |
| IND4 | KY052105 | India | Martins et al.,2017 |
| IND5 | KY052092 | North India | Martins et al.,2017 |
| IND6 | KY052098 | India | Martins et al.,2017 |
| IND7 | KY052100 | India | Martins et al.,2017 |
| IND10 | KY052095 | East India | Martins et al.,2017 |
| NEP1 | KY052099 | Nepal | Martins et al.,2017 |
| THA5 | KY052106 | Thailand | Martins et al.,2017 |
| THA6 | KY052097 | Thailand | Martins et al.,2017 |
| VIE5 | KY052115 | North C. Vietnam | Martins et al.,2017 |
| CHI2 | KY052110 | Yunnan China | Martins et al.,2017 |
| VIE10 | KY052113 | North C. Vietnam | Martins et al.,2017 |
| VIE9 | KY052111 | North C. Vietnam | Martins et al.,2017 |
| JAV1 | KY052139 | West Java | Martins et al.,2017 |
| JAV2 | KY052145 | Java | Martins et al.,2017 |
| JAV5 | KY052130 | East Java | Martins et al.,2017 |
| JAV6 | KY052134 | West Java | Martins et al.,2017 |
| JAV13 | KY052121 | West Java | Martins et al.,2017 |
| JAV14 | KY052138 | West Java | Martins et al.,2017 |
| JAV15 | KY052129 | Java | Martins et al.,2017 |
| BOR4 | KY052133 | Borneo | Martins et al.,2017 |
| BOR7 | KY052124 | Borneo | Martins et al.,2017 |
| LSI1 | KY052123 | Lesser Sunda Island | Martins et al.,2017 |
| LSI3 | KY052153 | Bangka Island | Martins et al.,2017 |
| BAL1 | KY052120 | West Bali | Martins et al.,2017 |
| SUM3 | KY052147 | Sumatra | Martins et al.,2017 |
| SUM4 | KY052136 | Sumatra | Martins et al.,2017 |
| SUM6 | KY052125 | Sumatra | Martins et al.,2017 |
| MAL1 | KY052118 | Peninsular Malaysia | Martins et al.,2017 |
| MAL2 | KY052119 | Peninsular Malaysia | Martins et al.,2017 |
| SRI1 | KY052116 | East Sri Lanka | Martins et al.,2017 |
| SRI2 | KY052117 | Sri Lanka | Martins et al.,2017 |

**Additional file: Table S2.** Samples details used for genetic analysis of red muntjac from India. n represents the sample size.

| **Origin** | | **Status** | **mtDNA** | | **Microsatellite** | |
| --- | --- | --- | --- | --- | --- | --- |
|  |  |  | **n** | **Types** | **n** | **Types** |
| *M. aureus* (Northwestern India) | | wild | 5 | Tissue | 18 | Tissue (15), Hairs (3) |
| *M. vaginalis* (Central India) | | wild | 3 | Tissue | 14 | Tissue (7), Antler (3), Hairs (4) |
| *M. vaginalis* (Northeastern India) | | wild | 2 | Tissue | 3 | Tissue (2), Bones (1) |
| *M. malabaricus* (Western Ghat India) | | wild | 4 | Tissue | 5 | Tissue |
| *M. vaginalis* (Andaman & Nicobar Islands) | | wild | 2 | Tissue | 2 | Tissue |

**Additional file: Table S3. Details of the primers used in this study to sequence complete mitogenome of red muntjac (**Hassanin et al. 2009**)**

| **Primer name** | **Primer Sequences** |
| --- | --- |
| DLU405 | 5' -ACCATGCCGCGTGAAACCAGCA-3' |
| 12SL41 | 5' -GYGYGGATRCTTGCATGTGTA-3' |
| U1230 | 5'-CACTGAAAATGCCTAGATGAG-3' |
| L2226 | 5'-CTAGGTGTAAACTAGRTGCTT-3' |
| 12SU829 | 5' -GCACGCACACACCGCCCGTCAC-3' |
| 16SL518 | 5' -CGCTTTCTTAATTGRTGGCTGC-3' |
| 16SU365 | 5' -AGCCTGGTGATAGCTGGTTGTCC-3' |
| 16SL1056 | 5' -AAGCTCCATAGGGTCTTCTCGTC-3 |
| 16SU946 | 5' -CCGTGCAAAGGTAGCATAATCA-3' |
| N1L64 | 5'-CCTAGNACTTTTCGTTCNACTA-3' |
| Uleu | 5' -GTGGCAGAGCCCGGTAATTG-3' |
| IleL | 5' -TTACTCTATCAAAGTAACTC-3' |
| N1U840 | 5' -TYCGAGCATCHTAYCCHCGATT-3' |
| N2L492 | 5'-TGGTTTAGBCCBCCTCAKCCYCC-3' |
| N2U354 | 5' -CACTTYTGAGTNCCAGAAGT-3' |
| AsnL | 5'-TAGGGTRTTTAGCTGTTAAC-3' |
| TrpU | 5' -AGACCAAGAGCCTTCAAAGC-3' |
| C1L339 | 5' -GCTTCWACTATDGADGATGC-3' |
| C1U246 | 5' -GGNGGNTTYGGHAAYTGACT-3' |
| C1L1017 | 5' -GAARATRAAGCCTAGRGCTCA-3' |
| C1U897 | 5' -TTYACHGTHGGAATAGAYGT-3' |
| C2L15 | 5' -GCRTCTTGRAANCCTARTTG-3' |
| SerU | 5' -CCCCCYAYWRYTGGTTTCAAGCCA-3' |
| A8L1 | 5' -GTKGAYGTRTCTAGTTGYGGCAT-3' |
| C2U603 | 5' -CAATGCTCHGARATYTGYGG-3' |
| C3L45 | 5' -GANARDGCTCCYGTDAGNGGTCA-3' |
| A6U654 | 5' -GCCTAYGTNTTYACYCTNCTAGT-3' |
| GlyL | 5' -TGATTGGAAGTCARYTGTAC-3' |
| C3U780 | 5' -GTHTCYATCTATTGATGAGG-3' |
| N4L27 | 5' -CAGGTYAGRGGDATDAGTAT-3' |
| U213M1 | 5' -AGCYTGYGAAGCAGCACTAGG-3' |
| L918M1 | 5' -GCKGTRGCTCCTATRTARCTTCA-3' |
| N4U840 | 5' - AGCTCHATYTGYYTHCGYCAAAC-3' |
| Leu2L | 5' - CCAATTTTTTGGYTCCTAAGRCC-3' |
| Ser2U | 5' -CCGAAAAAGYAYGCAAGAACTGC-3' |
| N5L652 | 5' -GCDGATTTTCCDGTKGCDGCTA-3' |
| N5U501 | 5' -GACGARCAGAYGCHAAYACAGC-3' |
| N5L1214 | 5' -GTDAKTADDAGGGCTCAGGCG-3' |
| N5U1146 | 5' -GGMAGCCTNGCNYTAACAGG-3' |
| N6RL154 | 50 -AGTTTAATGGDHTDGGDGATTG-3 |
| N6RU102 | 5' -CCATAACTRTAYAAAGCHGCAA-3' |
| CBL402 | 5' -CCTCARAATGATATTTGKCCTCA-3' |
| CBU162 | 5' -CAGGMCTATTCCTRGCHATACA-3' |
| LTHR | 5' -CCCTTYTCTGGTTTACAAGACC-3' |
| U1068 | 5' -CATCGGACAACTAGCATCTAT-3' |
| L482 | 5' -CCTGAAGWAAGAACCAGATG-3' |

**Additional file: Table S4. Details of used samples in the analysis**

| **S.No** | **S.ID** | **Localities** | **Microsatellites** | **Mitochondrial** |
| --- | --- | --- | --- | --- |
| 1 | BDHP1 | Himachal Pradesh, India | ✓ | ✓ |
| 2 | BDHP2 | Himachal Pradesh, India | ✓ | ✓ |
| 3 | BDPB1 | Punjab, India | ✓ | ✓ |
| 4 | BDUK1 | Uttarakhand, India | ✓ | ✓ |
| 5 | BDUK2 | Uttarakhand, India | ✓ | ✓ |
| 6 | BDUK3 | Uttarakhand, India | ✓ | NA |
| 7 | BDUK4 | Uttarakhand, India | ✓ | NA |
| 8 | BDUK5 | Uttarakhand, India | ✓ | NA |
| 9 | BDUK6 | Uttarakhand, India | ✓ | NA |
| 10 | BDUK7 | Uttarakhand, India | ✓ | NA |
| 11 | BDUK8 | Uttarakhand, India | ✓ | NA |
| 12 | BDUK9 | Uttarakhand, India | ✓ | NA |
| 13 | BDUK10 | Uttarakhand, India | ✓ | NA |
| 14 | BDUK11 | Uttarakhand, India | ✓ | NA |
| 15 | BDUK12 | Uttarakhand, India | ✓ | NA |
| 16 | BDUK13 | Uttarakhand, India | ✓ | NA |
| 17 | BDUK14 | Uttarakhand, India | ✓ | NA |
| 18 | BDUK15 | Uttarakhand, India | ✓ | NA |
| 19 | BDADM1 | Andaman, India | ✓ | NA |
| 20 | BDMP3 | Madhya Pradesh, India | ✓ | NA |
| 21 | BDAP2 | Arunachal Pradesh, India | ✓ | NA |
| 22 | BDADM1 | Andaman, India | ✓ | ✓ |
| 23 | BDADM2 | Andaman, India | ✓ | ✓ |
| 24 | BDOR1 | Odisha, India | ✓ | NA |
| 25 | BDOR2 | Odisha, India | ✓ | NA |
| 26 | BDOR3 | Odisha, India | ✓ | NA |
| 27 | BDOR4 | Odisha, India | ✓ | NA |
| 28 | BDAP1 | Arunachal Pradesh, India | ✓ | ✓ |
| 29 | BDMP1 | Madhya Pradesh, India | ✓ | ✓ |
| 30 | BDMP2 | Madhya Pradesh, India | ✓ | ✓ |
| 31 | BDNL1 | Nagaland, India | ✓ | ✓ |
| 32 | BDWB1 | West Bengal, India | ✓ | NA |
| 33 | BDWB2 | West Bengal, India | ✓ | NA |
| 34 | BDCG1 | Chhattisgarh | ✓ | ✓ |
| 35 | BDWB3 | West Bengal, India | ✓ | NA |
| 36 | BDUP1 | Uttar Pradesh, India | ✓ | NA |
| 37 | BDJK1 | Jharkhand, India | ✓ | NA |
| 38 | BDKRT1 | Karnataka, India | ✓ | ✓ |
| 39 | BDKRT2 | Karnataka, India | ✓ | ✓ |
| 40 | BDWG1 | Western Ghats, India | ✓ | ✓ |
| 41 | BDWG2 | Western Ghats, India | ✓ | ✓ |
| 42 | BDTN | Tamil Nadu, India | ✓ | NA |


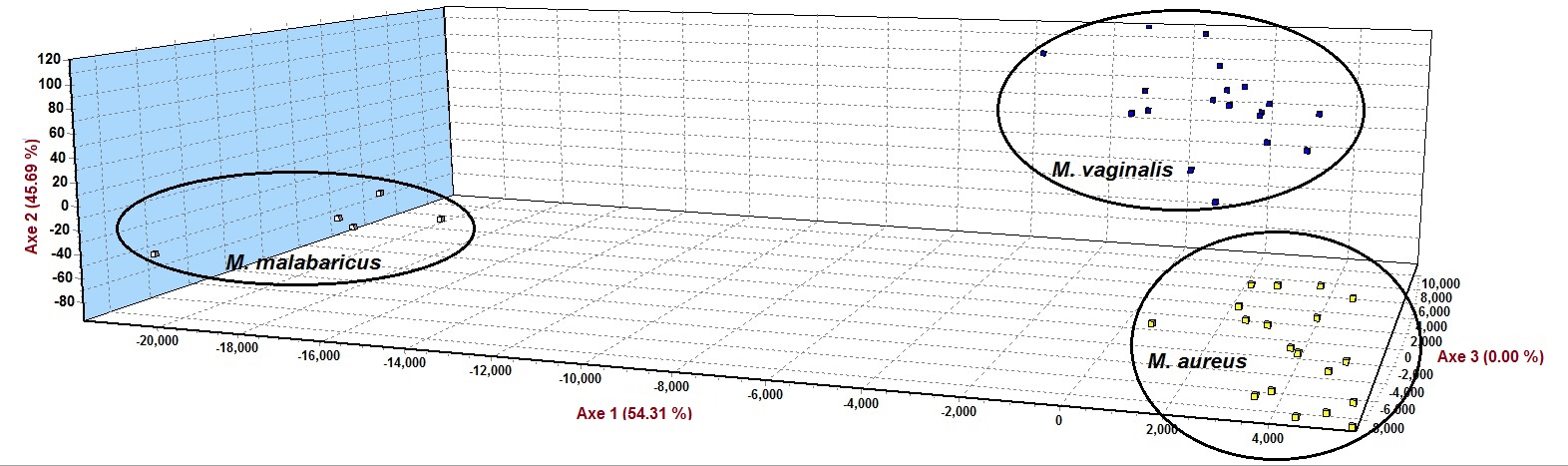


**Additional file: Figure S1.** Results of factor correlation analysis (FCA) using microsatellite markers indicating three major clusters in the Red muntjac population in India.
